# Le traitement chirurgical des fractures du cotyle: à propos de 22 cas

**DOI:** 10.11604/pamj.2014.17.123.2572

**Published:** 2014-02-21

**Authors:** Hicham Mahdane, Amine Elghazi, Mohamed Shimi, Abdelhalim Elibrahimi, Abdelmajid Elmrini

**Affiliations:** 1 Service de Chirurgie Ostéoarticulaire B4, CHU Hassan II, Fez, Maroc

**Keywords:** Fracture du cotyle, traitement chirurgical, ostéosynthèse, Acetabular fracture, surgical treatment, osteosynthesis

## Abstract

Vingt deux patients présentant une fracture incongruente de l'acétabulum ont été opérés et revues avec un recul moyen de 2 ans. Le but de cette étude est d'analyser les résultats fonctionnels et radiologiques des fractures de l'acétabulum après traitement chirurgical. La population était constituée de 17 hommes et 5 femmes, avec un âge moyen de 42,5 ans. Les fractures de l'acétabulum étaient répertoriées selon la classification de Judet et Letournel: 11 fractures de la paroi postérieure, une fracture de la colonne postérieure, six fractures transversales, quatre fractures transversales associées à une paroi postérieure, et une seule fracture de la colonne postérieure avec fracture de la paroi postérieure. Quatre patients présentaient des lésions associées du bassin, sept patients avaient une luxation postérieur de la hanche, deux une luxation centrale et deux avaient un traumatisme crânien associée. Deux voies d'abords ont été utilisées dans ce travail: la voie de Kocher Langenbeck (19 cas), et la voie de Dana Mears (4 cas). A partir du bilan radiologique initial (bassin face, ¾ alaire, ¾ obturateur et tomodensitomértie) on évaluer le déplacement, la congruence, tête/toit et tête/acétabulum ainsi que le degré de comminution. La qualité de réduction était évaluée selon les critères de Matta et les résultats fonctionnels selon la cotation de Merle D'Aubigné. Sur le Plan radiologique nous avons obtenu 56,52% de réduction anatomique, alors que sur le plan fonctionnel 78% patient avaient de bons et très bon résultats. Parmi les complications postopératoires, on a noté un seul cas d'infection cutanée superficielle, cinq ossifications héterotopiques. A distance un cas d'ostéonécrose aseptique de la tête fémorale et un cas de coxarthrose.

## Introduction

Nous rapportons notre expérience chirurgicale des fractures du cotyle d'après une série continue rétrospective de 22 cas. L'indication chirurgicale était posée devant la perte de la congruence articulaire entre la tête fémorale et l'acétabulum. C'est sur les radiographies du bassin (face, ¾ alaire et ¾ obturateur) bien décrites par Judet et Letournel [1] et surtout la tomodensitométrie que la congruence articulaire peut être appréciée. La TDM permet une analyse de la congruence dans le plan horizontal (tête/paroi), et dans le plan sagittal (tête/cotyle) grâce à l'apport des coupes de reconstructions coronales et sagittales. La parte de la congruence tête/toit était pour nous le facteur déterminant dans l'indication opératoire. De même, devant une hanche Potentiellement instable ou devant une incarcération fragmentaire le traitement était chirurgical. Le but de cette étude était d’évaluer les résultats fonctionnels et radiologiques du traitement chirurgical et l'indication des voies d'abord élargies notamment dans le traitement des fractures transversales.

## Méthodes

Vingt deux patients ont été opérés pour fractures de l'actétabulum (dont un patient présentant une fracture bilatérale du cotyle) entre 2009 et 2011 et revues avec un recul moyen de 2 ans. La population comprenait 17 hommes et 5 femmes, avec un âge moyen de 45 ans (<0 à 70 ans). Les circonstances du traumatisme étaient dominées par les accidents de la voie publique, les chutes d'un lieu élevé, puis les ensevelissements. Nous avons classés nos fractures selon la classification de Judet et Letournel avec: 11 fractures de la paroi postérieure, une fracture de la colonne postérieure, six fractures transversales, quatre fractures transversales associées à une paroi postérieure, et une seule fracture de la colonne postérieure avec fracture de la paroi postérieure (Tableau 1). Tous nos patients ont bénéficié un bilan radiologique préopératoire comportant une incidence de bassin de face et des incidences de ¾ alaire et obturateur ainsi qu'un examen tomodensitométrique du bassin entier.

**Tableau 1:** Le type de fracture selon JUDET ET LOUTERNEL

SIMPLE	Paroi postérieure (11 cas)
	Colonne postérieure (1 cas)
	Transversale (6 cas)
COMPLEXE	Colonne post + paroi post (1 cas)
	Transversale + paroi post (4 cas)

Dans notre série les lésions associées aux fractures de l'acétabulum sont rapportées sur le Tableau 2. A partir de ce bilan initial nous avons étudié la congruence verticale (tête/toit) et horizontale (tête/acétabulum) (Tableau 3) ainsi que la comminution fracturaire [2] qui était présente chez 45% des cas. Dans notre série un abord postérieur type Kocher-Langenbeck a été réalisé sur 18 hanches, la voie d'abord de Mears a été réalisé 4 fois, alors qu'un abord de Kocher-Langenbeck associée à une tronchantérotomie a été réalisée chez un seul patient.

**Tableau 2:** Les lésions associées au fractures du cotyle

Poly traumatisme	3 cas (13,6%)
Lésion du bassin	4 cas (18,1)
Luxation postérieure	7 cas (31,8%)
Luxation centrale	2 cas (9%)
Traumatisme crânien	2 cas (9%)
Paralysie sciatique	0 cas

**Tableau 3:** La congruence tête/toit et tête cotyle

Congruence TT		Congruence TC	
Congruence tête/toit	Congruence tête/acétabulum	Nombre de cas	Nombre de cas
Parfaite (TT3)	Parfaite (TC3)	0 hanche	3 hanches
Bonne (TT2)	Bonne (TC2)	2 hanches	6 hanches
Passable (TT1)	Passable (TC1)	8 hanches	8 hanches
Mauvaise (TT0)	Mauvaise (TC0)	13 hanches	6 hanches
**TT0 +TT1: 61% des cas**		**TC0 + TC1: 91,3% des cas**	

Tous nos patients ont été opérés sous anesthésie générale, l'installation s'est toujours faite sur table conventionnelle en décubitus latéral avec un aide au bout de la table, hanche maintenue en extension et genou fléchi à 90° afin de prévenir un étirement excessive sur le nerf sciatique. Le délai chirurgical moyen était de 6 jours (5 à 13 jours), une décharge au lit pendant une dizaine de jours était préconisée en postopératoire suivi d'une verticalisation son appui.

En postopératoire, les patients ont bénéficié d'un nouveau bilan radiologique complet permettant ainsi d'analyser la qualité de la réduction selon les critères de Matta et al [3]. Alors que les résultats fonctionnels ont été appréciés selon la cotation de Postel et Merle d'Aubigné [4].

## Résultats

### Réduction postopératoire

**Figure 1:**
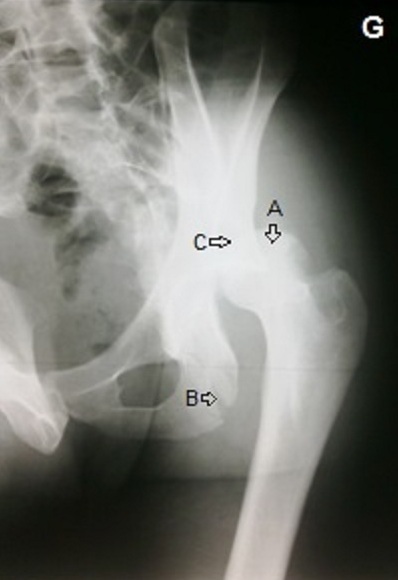
(Patient 1) radiographie de la hanche gauche en ¾ montrant une luxation postérieur de la tête fémoral (flèche A), une fracture de la colonne postérieure (flèche B) avec fracture de la paroi postérieure (flèche C) du cotyle gauche

**Figure 2:**
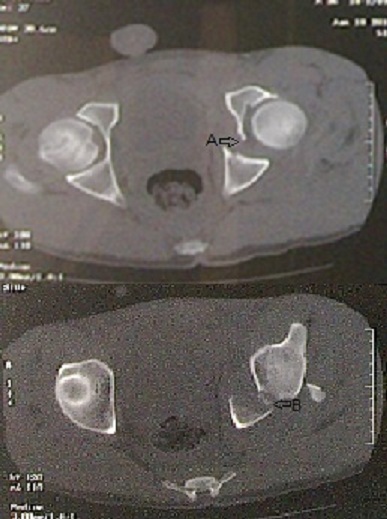
(Patient 1) coupe scannographique transversale montrant la fracture de la colonne postérieure avec fracture de la paroi postérieure (flèche A,B)

**Figure 3:**
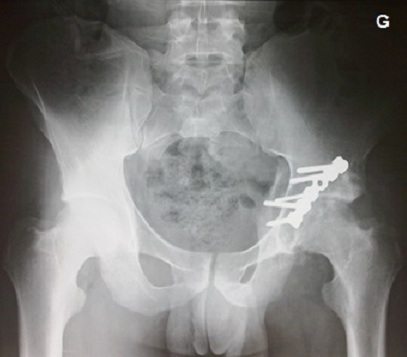
(Patient 1) Revue à 2 ans: réduction anatomique selon les critères radiologique de Matta, score Postel merle d'Aubigné à 17 à la révision

D'après les critères radiologiques de Matta et al [5], 56,52% des cas avaient une restauration anatomique de l'articulation et 36,6% des fractures avaient un résultat radiologique satisfaisant toutes classes confondues (Tableau 4). On rapporte un seul échec de réduction pour des fractures à composante transversale avec luxation centrale. Les fractures à composante postérieur avaient les meilleurs résultats avec 85% de réduction anatomique (Figure 1, Figure 2, Figure 3).

**Tableau 4:** Les résultats de la réduction selon les critères de Matta

La réduction (selon critères de Matta)	Nombre de cas	Fréquence en %
Anatomique (<1mm)	13 cas	56,52%
Satisfaisant (1 à 3mm)	7 cas	30,43%
Non satisfaisant (>3mm)	3 cas	13,05%
Total	23 hanches	100%

### Résultats fonctionnels

**Figure 4:**
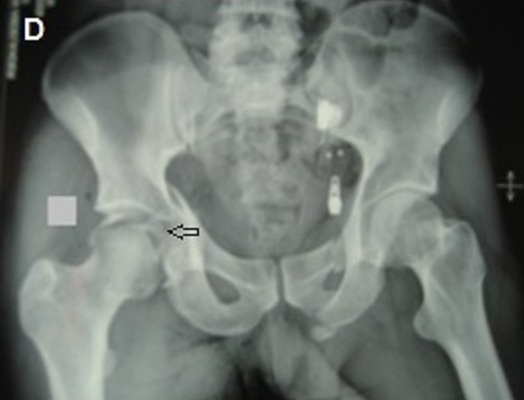
(Patient 2) radiographie du bassin de face montrant une fracture transversale associée à une fracture de la paroi postérieuredu cotyle gauche

**Figure 5:**
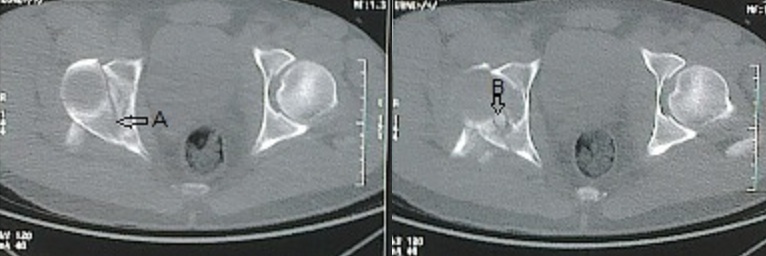
(Patient 2) coupe scannographique transversale qui montre la fracture transversale du cotyle droit avec la fracture de la paroi postérieure (flèche A,B)

**Figure 6:**
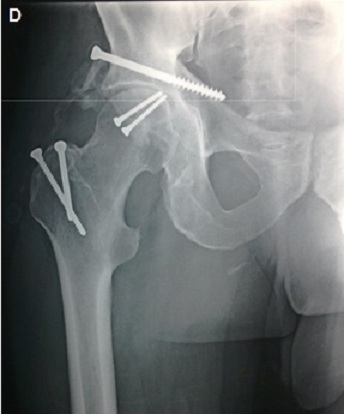
(Patient 2) revue à 2 ans: réduction anatomique selon les critères radiologique de Matta, score Postel Merle d'Aubigné à 17 à la révision

Au dernier recul 78% des patients avaient de bons, très bons et excellents résultats, nous avons constaté que la qualité des résultats fonctionnels était corrélée à la qualité de la réduction (Figure 4, Figure 5, Figure 6).

### Complications

Un seul cas d'infection cutanée superficielle à staphyloccoque aureus a été noté ayant bien évolué après reprise chirurgical de la cicatrice. Cinq patients présentaient des ossifications hétérotopiques, toutes classées stade I de Brooker [6] (100% des patients traités par voie élargie de Mears). Cependant la présence d'ossification n'altérait en rien le devenir fonctionnel de nos patients. Nous déplorons un seul cas de nécrose de la tête fémorale diagnostiquait à un an en postopératoire, chez un patient ayant présentait une luxation postérieur réduite tardivement. Sur les 23 cotyles opérés nous avant noté un seul cas de coxarthrose secondaire à la présence d'une vis intra-articulaire, avec bien sûr la réserve d'une courte série.

## Discussion

Nos résultats fonctionnels évalués selon le score de Merle d'Aubigné (77% de bons et excellents) sont superposables aux séries de la littérature: 76% de résultats bons à excellents pour la série de Judet et Letournel [1] et 80% pour la série de Matta et al [3]. Il en n'est de même sur le plan de la réduction, nos résultats anatomiques sont équivalents à ceux retrouvés par Duquennoy et al [2] en 1981 (60% de réduction anatomique toutes classes confondues), ainsi que Glas et Fessy en 2001 avec un taux de 61,6%.

Les voies d'abord et leurs indications en fonction du type anatomo-clinique de la fracture en bien était décrites par Judet et al [7]. Dans notre série nous avons toujours utilisé la voie de Kocher Langenbeck pour traiter les fractures de la paroi ou de la colonne postérieure, ainsi que les fractures à composante transversale juxta ou infratecale, alors que pour les fractures transtecales, la voie élargie s'impose afin d'obtenir un meilleur contrôle endoarticulaire de celui-ci [8], dans notre expérience nous avons utilisé la voie de Mears chez 4 patients.

Les ossifications hétérotopiques sont des complications fréquentes de la chirurgie de l'acétabulum et leur incidence varie de 30 à 70% selon les séries [9].

Dans notre étude le taux d'ossifications héterotopiques était de 23%, toutes des stades I de Brooker, avec les réserves d'une courte série. Dans la littérature, l'incidence de l'ostéonécrose aseptique de la tête fémorale varie de 2 à 10% [9]. Letournel et Judet rapportent [10] 3,8% d'ostéonécose de la tête fémorale et ne trouvent pas de corrélation avec le délai de réduction de la luxation de la tête fémorale. Dans notre série, un seul patient a développé une ostéonécrose de la tête fémorale.

L'arthrose post-traumatique est de loin la complication la plus fréquente, avec une incidence qui varie entre 20 et 50%. Louternel et Judet [10] ont trouvé 10% d'arthrose lorsque la réduction est parfaite et 36% devant des réductions imparfaites. L'incidence rencontrée dans la série de Glas et al [11] lors des réductions anatomiques est de 2,5% alors quelle est de 27,3% lors de la réduction non anatomique. Il existe bien un consensus sur le faite que la meilleure prévention contre la survenue d'arthrose soit l'obtention de la réduction la plus anatomique possible.

Dans notre série, un seul cas d'arthrose post-traumatique a été rapporté à 1 an en postopératoire secondaire à une vis intra-articulaire, sous les réserves d'un recul faible de 3 ans.

## Conclusion

Les critères pronostiques majeurs des fractures de l'acétabulum sont la qualité de réduction et la congruence tête/toit obtenue en postopératoire, du faite, les fractures complexes nous incitent à utiliser des voies d'abords élargies permettant un meilleur contrôle de la réduction. L'ensemble de nos résultats radiologiques et fonctionnels nous encourage à poursuivre le traitement chirurgical des fractures incongruentes de l'acétabulum à l'exception des fractures très comminutives dont la réduction devient incontrôlable
